# The Role of Lifestyle Factors in Controlling Blood Pressure among Hypertensive Patients in Two Health Facilities in Urban Ghana: A Cross-Sectional Study

**DOI:** 10.1155/2020/9379128

**Published:** 2020-09-07

**Authors:** Emefa Modey Amoah, Darlene Esinam Okai, Adom Manu, Amos Laar, Joseph Akamah, Kwasi Torpey

**Affiliations:** ^1^University of Ghana School of Public Health, Accra, Ghana; ^2^University of Ghana Medical School, Accra, Ghana

## Abstract

**Introduction:**

Despite efforts to combat hypertension by pharmacotherapy, hypertension control rates remain low. Lifestyle modifications of individuals diagnosed with hypertension have prospects for the prevention and control of hypertension. This study assessed the effect of modifiable lifestyle factors on blood pressure control among adults in urban Accra.

**Methods:**

In this cross-sectional study, 360 diagnosed hypertensive patients who were ≥18 years old, selected from two secondary-level referral hospitals in the Greater Accra Region, were interviewed. Demographic information, diet components, and exercise assessments as well as blood pressure measurements were taken. Chi-squared tests and binomial logistic regression were used to determine the association between demographic and lifestyle factors with blood pressure control. Area under the receiver-operator curves (AUROC) was used to identify lifestyle factors predicting optimal blood pressure control among patients diagnosed with hypertension.

**Results:**

Approximately 54.2% of participants had no knowledge of either causes or complications of hypertension. Similarly, 52.5% of patients that had not achieved blood pressure control lacked knowledge of causes or complications of hypertension. Longer time since diagnosis of 2–5 years (AOR = 0.08 (95% CI: 0.01–0.47)) and 6–10 years (AOR = 0.08 (95% CI: 0.01–0.50)) and diets, mainly composed of meat (AOR = 0.13 (95% CI: 0.02–0.70)) and starch (AOR = 0.14 (95% CI: 0.03–0.79)), predicted poor blood pressure control compared to patients diagnosed within a year and diets without meat and starch as main components, respectively. Additionally, engaging in some physical activity of 30 minutes to one hour (AOR = 5.64 (95% CI: 2.08–15.32)) and more than an hour (AOR = 11.38, 95% CI: 2.01–64.47)) predicted blood pressure control.

**Conclusion:**

The study concludes that increased physical activity, abstaining from alcohol and smoking, increased intake of fruits and vegetables, and reduced intake of carbohydrates, meat, and fat have a positive influence on blood pressure control. Lifestyle modifying factors have a key role in complementing pharmacotherapy in hypertension control.

## 1. Introduction

Hypertension is universally acknowledged as a preventable and modifiable risk factor for cardiovascular disease [1]. It is the leading global risk factor for death and disability among all ages [2]. Hypertension has duly been acknowledged as a global and public health crisis by the World Health Organization (WHO) in 2013 [3–5]. The global push for the reduction of this risk factor has resulted in targets for a substantial reduction in elevated blood pressure worldwide [6]. Among the set of Voluntary Global Targets to be achieved by the year 2025, hypertension is expected to be reduced or controlled by 25%. Additionally, a 10% to 30% reduction is expected in behavioural or lifestyle factors that influence hypertension.

The increasing prevalence of hypertension in developed and developing regions is attributed to a number of factors. These include population growth and ageing, changes in diet, physical inactivity, increasing body mass index (BMI), and harmful use of alcohol [4]. In developing country settings, the prevalence of hypertension within urban areas has been linked to urbanization and the associated lifestyle changes such as high consumption of salt, alcohol, and fats and lowered exercise or physical activity [3, 7–9]. In Ghana, the prevalence of hypertension was estimated at 13% among men and women [10, 11]. The influence of urbanization on hypertension in Ghana is evident in recent findings from a multicentre hospital study that shows poor blood pressure control predominantly among urban dwellers [12]. Earlier studies among urban communities in Accra have shown low levels of physical activity among both males and females [13].

Among patients diagnosed with hypertension, the initiation and adherence to pharmacological treatment have been proven to help individuals attain optimal blood pressure (BP) control [2]. Nonetheless, despite the available and effective antihypertensive medications, blood pressure control remains elusive [7, 14]. Uncontrolled hypertension contributes to the burden of cardiovascular diseases (CVDs). The low levels of control are compounded by equally low rates of awareness and treatment [7]. Where medication-led control is suboptimal, lifestyle modification is critical to control blood pressure either alongside or independently [15]. Comprehensive interventions which include lifestyle modifications have been suggested to successfully control BP levels and reduce deaths and disability from CVDs [16, 17].

Control rates of hypertension vary widely across and within populations. Various studies have reported rates of 1%–49% [7, 18–21]. Control rates in developing countries are relatively low due to weak health systems [4]. In Ghana, control rates range from 3.5% to 42% [11–13]. The national control rates in Ghana by sex show that 17% of women and 6% of men on treatment report blood pressure control [10]. Several individual and background factors such as sex, age, education, pill burden, comorbidity, eating habits such as high salt consumption, alcohol, tobacco, body mass index, and exercise have been identified as factors associated with blood pressure control [5, 12, 17, 22–24]. The Community-based Hypertension Improvement Project (ComHIP) in Ghana assessed the use of routine drug therapy and supplemented with nondrug therapy (lifestyle modification) and reminder systems among hypertensive patients. The results showed a higher level of blood pressure control of 72% after a year [25].

The identification of lifestyle factors associated with blood pressure control is necessary for improved chances of BP control outside of pharmacological treatment. Of the lifestyle modifications believed to contribute to both the prevention of hypertension and its control, dietary modification has been emphasized in several studies [4, 7, 13, 17]. Bad dietary habits have contributed to the increased hypertension prevalence in many developing countries including Ghana [3, 14, 26].

The multifactorial nature of risk factors implies that preventive efforts are needed at patient, physician, and health system levels to reduce hypertension [27]. The evidence suggests that modest improvements in more than one risk factor can contribute to an overall reduction [28]. This has paved the way for multivariable risk prediction to estimate hypertension and CVD risk from multiple risk factors in the USA, Iran, Korea, and China for different populations [29, 30]. This has facilitated the identification of high-risk individuals for timely and targeted interventions.

Beyond the prediction of high-risk individuals likely to develop hypertension, monitoring BP control is equally of value. In spite of the crucial role of lifestyle modification in controlling blood pressure, very limited evidence exists in Ghana on the extent to which lifestyle factors influence blood pressure control. This study aimed to identify the factors that influence hypertension control in adults on antihypertensive medication in urban Ghana. The purpose of this is to develop a lifestyle-based prediction model for hypertension control in a population of urban Ghanaian adults. The lifestyle factors measured in the study included physical activity, alcohol consumption, tobacco use, and diet.

## 2. Materials and Methods

### 2.1. Study Design and Setting

This was a descriptive, cross-sectional study at the outpatient hypertension clinics in two secondary-level referral hospitals in the Greater Accra Region of Ghana. Both hospitals serve urban populations and run hypertensive clinics on two days of the week.

### 2.2. Study Sites Characteristics

La Dadekotopon is an entirely urban municipality. It has an area of 36.3sqkm with a population of 183500. The household size is 3.6 persons/household. Malaria is the leading communicable disease in the municipality. Hypertension accounts for 20% of noncommunicable diseases. LEKMA Hospital is situated in the Ledzokuku Krowor Municipal Assembly. The Assembly has an area of 47.58sq.km with a population of 227,932. It has a household size of 2.8 but the average population per house is 10.4. Upper respiratory tract infections and malaria are the leading cause of communicable diseases. Hypertension is the commonest noncommunicable disease [31, 32].

### 2.3. Participants

A total of 360 participants were interviewed in this study.

### 2.4. Inclusion and Exclusion Criteria

Participants were eligible for the study if they were 18 years and older. They also needed to have been diagnosed as hypertensive with a systolic blood pressure (SBP) of at least 140 mm Hg and/or a blood pressure (DBP) of at least 90 mm Hg. They must have also been on antihypertensive treatment for no less than a year. Patients with coexisting medical conditions such as diabetes were included in the study once they met a blood pressure reading of or at least 130 mm Hg (SBP) and/or a blood pressure (DBP) of at least 80 mm Hg if they were diabetic.

Patients with psychiatric conditions or previous hypertensive emergency as well as those on medication that affected blood pressure were excluded from the study. Pregnant women were also excluded from the study.

### 2.5. Sample Size

The sample size was estimated under the following assumptions: *z* = 1.96, *d* = 0.05, prevalence of adherence to hypertension medication of 0.30 based on a study of urban adults in Accra [33]. The calculated minimum sample size required was 323.

### 2.6. Sampling

A list of hypertensive patients given appointments for each day was obtained from the records department. Numbers were assigned to the individuals on the list. This list was deidentified and identification numbers were assigned to the individuals on the list to control for sampling bias. The numbers were randomly selected using Excel random number generation. After the selection, the names of individuals on the list corresponding to the selected numbers were contacted after they had seen the doctor to conduct the interview. An average of 50 to 60 patients was booked for each clinic day for both facilities with the facility with a higher caseload contributing 30–35 per day. In total, 220 participants were recruited from LEKMA and 140 were recruited from La Dadekotopon and the sample for each facility was proportional to size.

### 2.7. Data Collection and Measures

A written informed consent (signed or thumb-printed) was obtained from each participant. Illiterate respondents nominated an independent witness to attest to the consent process. Consent was sought from participants by trained researcher after the study was explained to them. Participants were interviewed after they had completed their doctor's visit. Data was collected using an interviewer administered structured questionnaire composed of five [5] different sections.

The first section of the questionnaire recorded a minimum of two [2] and a maximum of five [5] BP readings inclusive of previous visits to the hospital. This section was completed after accessing this information from the individual patient folders. The BP readings recorded were taken by the healthcare provider as part of the routine care provided at the clinic. Blood pressure readings were taken in the nondominant arm using an automated sphygmomanometer with the patient in an upright sitting position after having rested for at least 10 minutes. Blood pressure classification for patients with more than one visit was based on the averaging of 2–5 recorded readings [17, 34]. Hypertension was defined as a diastolic reading of 90 mm Hg or more and a systolic of 140 mm Hg or more. Participants were categorized as having controlled blood pressure based upon this threshold.

The second section consisted of questions on the sociodemographics of participants including age in single years, sex of participant, level of education, and area of residence. Additional information on the participant's health and treatment collected information on the number of pills being taken and the presence of comorbid conditions. Comorbidities reported were verified from patient records.

The third section examined patients' knowledge about hypertension, its causes, and complications as well as comorbidities and medications. A final knowledge score was obtained for each respondent. The respondents were scored on their knowledge of each domain: normal adult blood pressure and the number of causes of hypertension and complications of hypertension they knew. Scores of 0 represented no knowledge of either the causes or complications of high blood pressure. 1 represented knowledge of either the causes or the complications and scores of 2 represented those who knew both causes and complications of hypertension. Participants were considered as having “Good knowledge” for a score of 2, as “Moderate knowledge” for a score of 1, and “No knowledge” for a score of 0.

In the final section, questions that measured lifestyle factors such as weekly exercise by duration, alcohol consumption in units, tobacco use and smoking history, and dietary habits were posed. Dietary components were assessed by a self-reported response of yes or no to the question “Is your diet largely based on Meat and fatty foods?” Similarly, the question was posed also for starchy foods, fruits, and vegetables, salt, or any others.

### 2.8. Data Analysis

Frequencies and proportions of the background factors by categories were done to describe the sample and show distribution. Means of continuous outcomes, age, and alcohol units consumed were estimated. The *t*-test was used to compare means for continuous variables: age and alcohol in units. Cross-tabulations of client sociodemographic and lifestyle factors influencing hypertension control were undertaken. Descriptive analysis was conducted using Pearson's *χ*^2^ and Fishers exact test were appropriate. The hypertension control status of each client was determined by calculating the average of all SBP and DBP readings recorded. Blood pressure readings of 140/90 mmHg or less were classified as optimal control. Blood pressure control was coded as 0 for poor control and 1 for optimal control.

Binary logistic regression model was used in assessing the influence of lifestyle factors on hypertension control. In estimating the predictive effect of lifestyle factors on blood pressure control among patients recruited in the study, two nested models were fitted. Model 1 consists of only lifestyle factors and model 2 integrated both the lifestyle factors and sociodemographic characteristics. The performance of these two models was evaluated by the area under receiver-operating characteristic curve (AUROC) and Akaike information criterion (AIC). Background variables that were controlled for in the analysis were sex, age, education, knowledge, presence of comorbidity, and number of pills taken. All analyses were conducted in STATA and statistical significance was set at a 95% confidence level.

### 2.9. Ethical Considerations

Ethical clearance was sought from the Ghana Health Service Ethics Review Committee with approval number GHS-ERC: 037/12/17. Permission to conduct the research at Ghana Health Service facilities was also sought from the Greater Accra Regional Health Directorate and subsequently from the Municipal Health Directorates and Heads of both facilities.

## 3. Results

Among the sample of patients interviewed, females constituted a larger proportion (70%). The mean age of participants was 61.9 ± 0.6 years with ages ranging from 27 to 94 years. The average time since diagnosis for patients in this study was 7.6 ± 0.3 years. Among the 51% of participants who consumed alcohol, less than a unit of alcohol was consumed weekly and smoking history was reported in a few patients (8.9%). Less than half of the patients in this study were engaged in any form of weekly exercise activity, and carbohydrates were a major component of the diets reported (91%). Further details of the background characteristics of the participants are available in Table 1.

Assessing patient knowledge shows that approximately 54.2% of patients did not have any knowledge of hypertension or its causes and complications (Table 1). Similarly only 17% of patients had accurate knowledge of hypertension.

Table 2 summarizes the sociodemographic characteristics of patients who have been on treatment against their hypertension control status. Approximately 23% of patients assessed had achieved blood pressure control. Age categories, sex, time since diagnosis, presence of comorbidity, number of pills taken, history of smoking, duration of exercise, and carbohydrate, meat, and vegetable components of diets were significantly associated with blood pressure control (*p* < 0.05). Among patients with uncontrolled blood pressure, 52.5% did not have any knowledge on hypertension or its causes and complications (Table 2). Approximately 59.1% of patient with uncontrolled blood pressure consumed alcohol, and 66% did not engage in any form of exercise. By dietary components, approximately 96.4% report diets with carbohydrates constituting a major component and 80% do not have fruits as a major component of their meals. Nonetheless, among participants that achieved blood pressure control, 90% reported that meat and oils were not a major component of their diets (Table 2).

### 3.1. Predictors of Blood Pressure Control

**Figure 1:**
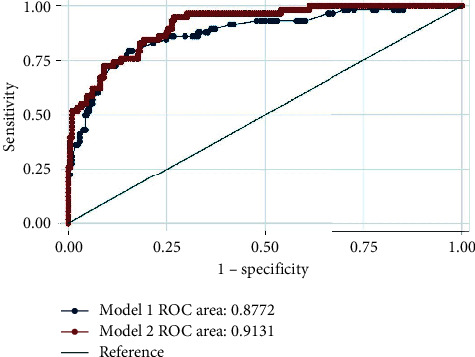
ROC comparing the performance of two models used in assessing the effect of lifestyle factors in predicting blood pressure control among urban adults.

To assess the lifestyle factors on blood pressure control among urban adults, two nested models were fitted. No significant difference in AUROC between the two nested models was observed; model 2, however, performed better with a larger area (AUROC = 0.91, 95% CI: 0.87–0.95).  Table 3 presents the output of the two models and Figure 1 graphically illustrates the performance of the models. After controlling for background factors (age, sex, education, knowledge, comorbidity, and number of BP pills taken), 2–5 yrs since diagnosis (*p*=0.005), 6–10 yrs since diagnosis (*p*=0.006), and diets with meat (*p*=0.017) and starch (*p*=0.025) as their main components predicted lower blood pressure control compared to patients diagnosed under a year and with diets without meat and starch as main components, respectively.

Physical activity between 30 minutes to an hour (*p* < 0.001) and more than one hour (*p*=0.006) predicted blood pressure control when controlling for sociodemographic and other lifestyle factors (Table 3). The odds of having blood pressure controlled were about 5 times higher among patients engaging in up to one hour of exercise weekly compared to patients who did not engage in any exercise activity (AOR = 5.64, 95% CI: 2.08–15.32). Among patients who engaged in 30 minutes to 1 hour of exercise activity, the odds of having blood pressure controlled were about 11 times compared to patients who did not engage in any exercise (AOR = 11.38, 95% CI: 2.01–64.47) (Table 3).

Patients that have lived with hypertension for 2–5 years were found to have a reduction in the odds of having their blood pressure controlled compared to those that have been diagnosed within a year (AOR = 0.08, 95% CI: 0.01–0.47). Similarly, among those diagnosed over 6–10 years, the odds of controlled blood pressure were reduced (AOR = 0.08, 95% CI: 0.01–0.5).

## 4. Discussion

In this study, one out of every five patients on antihypertensive medication was able to attain control of their blood pressure—a finding consistent with the range of control estimated from other studies in Ghana [11–13]. The fact that a larger proportion of patients on antihypertensive medication, however, are unable to control their blood pressure is a worrying issue. Sarfo et al. [12] and Obirikorang et al. [35] similarly reported that patients diagnosed over a longer period more often have poor blood pressure control. This highlights a potential predisposition of prolonged duration of the disease to a lowered motivation to control the disease and low perceived severity of the condition in such patients [35]. This poor observance has been reported as an important contributor to inconsistencies in blood pressure control across different settings [36]. This nonetheless emphasizes an aspect of blood pressure control that necessitates timely intervention. It is consequently important to encourage patients particularly as they progress through the course of the disease to commit to treatment and lifestyle recommendations to ease the burden of uncontrolled blood pressure. Such encouragement is most critical during the first year of diagnosis particularly as control reduces among patients from 2 years onwards since the time of diagnosis.

This study found that more than half of participants did not have accurate knowledge of hypertension—a finding in support of evidence linking poor knowledge of hypertension to suboptimal blood pressure control [37–39]. This nonetheless highlights the potential of improving knowledge of patients on hypertension, its causes, blood pressure targets, and the lifestyle modifications necessary to manage the condition. This is essential particularly among diagnosed hypertensive patients to enable them to attain and possibly maintain control of their blood pressure early within the first year of diagnosis.

Weekly exercise, comprising exercise activity of up to an hour or more, was identified to contribute to blood pressure control among diagnosed patients assessed. This finding is encouraging as evidence continually shows that the frequency of exercise is a significant predictor of hypertension control [13, 14, 16, 26]. The fact that more than half of patients in this study did not engage in any exercise activity is a reflection of an earlier report of low levels of physical activity among Ghanaian urban adults [13]. It is vital that urban patients are encouraged to engage in more exercise to attain blood pressure control. Although the type or level of intensity of the exercise was not measured in this study, the findings lend support to the current global recommendation of accumulating a total of 150 minutes of moderate-intensity aerobic physical activity per week or 75 minutes of vigorous-intensity aerobic physical activity for adults up to age 64 for improved health outcomes [40].

In assessing the components of an individual's diet, the findings of this study indicate that patients with poor blood pressure control reported diets mainly composed of meats, fats, and starch. These are dietary components identified as not supportive of reducing blood pressure [4, 14, 16]. The findings of this study show the association of meaty and fatty diets with uncontrolled blood pressure among Ghanaian adults—a discovery noted in an earlier study to be associated with increased blood pressure levels in Ghana [41]. Our study has some limitations. Even though correlations were identified between starch, meats, and fats as main components of the typical diets assessed, a standard dietary assessment tool was not employed. Further studies should utilize such tools in dietary assessment among hypertensive patients to ensure comparability. Although hypertension has been shown to be linked with body mass index (BMI), the second limitation of this study is that BMI and obesity status were not measured.

Patient counselling has been shown to reinforce the importance of adhering to diets rich in fruits and vegetables and fat products [42]. This becomes essential in improving patient knowledge for the successful management and control of blood pressure in urban adults. Regardless of diets being predominantly composed of starch, they were low in sodium, meat, and fats. These patients have likely begun lifestyle changes to sodium, meat, and fat intake to control their blood pressure effectively. It is important to note, however, that, among this sample of patients, vegetable components were low—an outcome requiring further assessment for appropriate intervention.

Within this population of urban adults, alcohol consumption also did not have a significant effect on blood pressure control. This finding is at variance with other studies of alcohol use and hypertension in Ghana [35]. It is plausible that, in this population, alcohol consumption reported was relatively lower and thus not of significant influence. This finding nonetheless reflects the national progress reported by the 2014 Ghana Demographic and Health Survey of reducing levels of alcohol and tobacco use among individuals diagnosed with hypertension [10].

This study demonstrates that a variety of lifestyle factors can reflect the hypertension control status of an adult patient. The findings of this study particularly show that modifiable factors, such as duration of exercise, alcohol intake, and dietary composition, were associated with BP control in patients on antihypertensive medication. These results are in agreement with the identified contribution of diet and exercise to blood pressure control in both men and women [4, 16, 43] and particularly among those on antihypertensive medication [24]. These findings signify the role of monitored diet and exercise in alliance with medication to achieving blood pressure control in urban adult patients.

## 5. Limitations

Our study is not without limitations. Even though correlations were identified between starch, meats, and fats as main components of the typical diets assessed, a standard dietary assessment tool was not employed. Further studies utilizing such tools in dietary assessment among hypertensive patients would ensure comparability.

A second limitation is that responses to exercise were subjective and self-reported. However, the use of well trained research assistants and to explain duration and intensity required for categorization ensured that responses obtained were as accurate as possible.

## 6. Conclusion

The study concludes that, among patients diagnosed and on antihypertensive medication, increased physical activity, abstaining from alcohol and smoking, increased intake of fruits and vegetables, and reduced intake of carbohydrates, meat, and fat have a positive influence on blood pressure control. Lifestyle modifying factors have a key role in complementing pharmacotherapy in hypertension control. Lifestyle modifying factors should be an integral part of management of patients with hypertension.

## Figures and Tables

**Table 1:** Background characteristics of respondents.

Characteristic	Frequency (*n*)	Percent (%)
Age		
27–45	19	5.28
46–55	69	19.17
56–65	150	41.67
66–94	122	33.89

Sex		
Male	105	29.17
Female	255	70.82

Education		
No formal education	92	25.56
Primary	109	30.28
Junior high	85	23.61
Secondary	57	15.83
Tertiary	17	4.72

Knowledge		
No knowledge	195	54.17
Moderate knowledge	104	28.89
High knowledge	61	16.94

Length of diagnosis		
1 year	33	9.17
2–5 years	133	36.94
6–10 years	116	32.22
>10 years	78	21.67

Comorbidity		
No	109	30.28
Yes	251	69.72

Number of pills		
1.2	131	36.39
2–4	229	63.61

History of smoking		
No	328	91.11
Yes	32	8.89

Alcohol consumption		
No	177	49,17
Yes	183	50.83
Alcohol consumption (in units)^∗^	0.72 ± 0.83	

Duration of exercise per week		
None	209	58.06
30 min–1 hr	125	34.72
>1 hour	26	7.22

Major component in diet		

Carbohydrates		
No	31	8.61
Yes	329	91.39

Meat and fats		
No	213	74.22
Yes	74	25.78

Fruits and vegetables		
No	203	71.23
Yes	82	28.77

High sodium		
No	250	93.98
Yes	16	6.02

^∗^ *n* is the 183 patients who indicated alcohol consumption.

**Table 2:** Association between lifestyle characteristics and blood pressure control.

Variable	Controlled BP		
	No *n* (%)	Yes *n* (%)	*p* value
Nonmodifiable Factors			
Mean age (±SD)	61.2 ± 0.61	64.1 ± 1.31	**0.012**
Age categories			**0.031**
27–45	15 (5.43)	4 (4.76)	
46–55	53 (19.20)	16 (19.05)	
56–65	125 (45.9)	25 (29.76)	
66–94	83 (30.07)	39 (46.43)	
Sex			**0.009**
Male	90 (85.71)	15 (14.29)	
Female	186 (72.94)	69 (27.06)	
Educational*∗*			0.077
No formal education	74 (80.43)	18 (19.57)	
Primary	90 (82.57)	19 (17.43)	
Junior high	56 (65.88)	29 (34.12)	
Secondary	43 (75.44)	14 (24.56)	
Tertiary	13 (76.47)	4 (23.53)	
Knowledge			0.056
No knowledge	145 (52.54)	50 (59.52)	
Moderate knowledge	77 (27.90)	27 (32.14)	
High knowledge	54 (19.57)	7 (8.33)	
Comorbidity			**<0.001**
No	69 (25)	40 (47.62)	
Yes	207 (75)	44 (52.38)	
Number of pills			**<0.001**
1.2	85 (30.80)	46 (54.76)	
2–4	191 (69.20)	38 (45.24)	
Time since diagnosis			
1 year	21 (7.61)	12 (14.29)	**0.008**
2–5 years	112 (40.58)	21 (25.00)	
6–10 years	91 (32.97)	25 (29.76)	
>10 years	52 (18.84)	26 (30.95)	
History of smoking			**0.017**
Yes	30 (10.87)	2 (2.38)	
No	246 (89.13)	82 (97.62)	
Lifestyle factors			
Alcohol consumption			**<0.001**
No	113 (40.94)	64 (76.19)	
Yes	163 (59.06)	20 (23.81)	
Alcohol use (per unit)	0.85 ± 0.83	0.31 ± 0.67	**<0.001**
Duration of exercise/week			**<0.001**
None	183 (66.30)	26 (30.95)	
30 mins–1 hr	82 (29.71)	43 (51.19)	
>1 hour	11 (3.99)	15 (17.86)	
Major component in diet			
Carbohydrates			**<0.001**
No	10 (3.62)	21 (25.0)	
Yes	266 (96.38)	63 (75.0)	
Meat and fats			**0.001**
No	157 (69.78	56 (90.32)	
Yes	68 (30.22)	6 (9.68)	
Fruit and vegetable			**<0.001**
No	177 (81.19)	26 (38.81)	
Yes	41 (18.81)	41 (61.19)	
High sodium*∗*			0.120
No	193 (92.79)	57 (98.28)	
Yes	15 (7.21)	1 (1.72)	
Total	**276 (76.67)**	**84 (23.33)**	

*∗*Cell count less than 360 due to nonresponse ^a^based on Fischer's exact test.

**Table 3:** Models used in assessing the effect of lifestyle factors on blood pressure control among urban adults.

	Model 1: adjusted effect of lifestyle factors on blood pressure control without controlling for demographic factors			Model 2: adjusted effect of lifestyle factors on blood pressure control when controlling for demographic factors		
	AOR	95% CI	*p* value	AOR	95% CI	*p* value
Time since diagnosis						
1 year	1.00			1.00		
2–5 years	0.17	0.04–0.67	0.012	**0.08**	**0.01–0.47**	**0.005**
6–10 years	0.14	0.03–0.56	0.006	**0.08**	**0.01–0.5**	**0.006**
>10 years	0.42	0.11–1.63	0.212	0.25	0.04–1.58	0.139
History of smoking						
Yes	1.00			1.00		
No	1.95	0.34–11.1	0.451	3.09	0.38–25.5	0.294
Units of alcohol per week	0.47	0.25–0.9	0.023	0.61	0.31–1.21	0.158
Activity						
No activity	1.00			1.00		
30 mins–1 hour	4.67	2.06–10.58	<0.001	**5.64**	**2.08–15.32**	**0.001**
≥1 hour	7.91	1.87–33.45	0.005	**11.38**	**2.01–64.47**	**0.006**
Major component in diet						
Starchy: yes	0.18	0.05–0.73	0.017	**0.14**	**0.03–0.79**	**0.025**
Meat and fat: yes	0.13	0.03–0.66	0.014	**0.13**	**0.02–0.7**	**0.017**
Fruit and vegetables: yes	2.18	0.89–5.38	0.089	2.54	0.9–7.2	0.080
Model performance indices						
AUROC (95% CI)	87.72% (82.39–93.06)			91.31% (87.47–95.16)		
AIC	202.57			194.92		

AOR: adjusted odds ratio, CI: confidence interval, ref: reference category, ^∗^*p* < 0.05, ^∗∗∗^*p* < 0.001, and ^∗∗∗^*p* < 0.001. Demographic factors controlled: age, sex, education, knowledge, comorbidity, and number of pills taken. AUROC: area under the receiver-operating characteristic curve. AIC: Akaike's information criterion.

## Data Availability

The datasets analysed during the current study are available from the corresponding author on reasonable request..
